# Prognostic factors for histiocytic and dendritic cell neoplasms

**DOI:** 10.18632/oncotarget.21920

**Published:** 2017-10-19

**Authors:** Joji Shimono, Hiroaki Miyoshi, Fumiko Arakawa, Kensaku Sato, Takuya Furuta, Reiji Muto, Eriko Yanagida, Yuya Sasaki, Daisuke Kurita, Keisuke Kawamoto, Koji Nagafuji, Koichi Ohshima

**Affiliations:** ^1^ Department of Pathology, Kurume University, School of Medicine, Kurume, Japan; ^2^ Department of Hematology, Kurume University, School of Medicine, Kurume, Japan; ^3^ Department of Hematology, Hokkaido University Graduate School of Medicine, Sapporo, Japan

**Keywords:** dendritic cell neoplasm, histiocytic sarcoma, survival, prognostic factor, BRAF

## Abstract

Histiocytic and dendritic cell neoplasms are rare and poorly studied. We report the clinical characteristics and prognostic factors in such cases in Japan. We investigated the clinical characteristics and survival in **87** adult patients with histiocytic and dendritic cell neoplasms. Fifty patients had histiocytic sarcoma, 12 had Langerhans cell histiocytosis, 11 had follicular dendritic cell sarcoma, 8 had Langerhans cell sarcoma, 6 had interdigitating cell sarcoma and 1 had indeterminate dendritic cell sarcoma. The median follow-up period was 18.0 (range: 9.6-71.8) months, and median overall survival (OS) was **23.5 months**. The 2-year OS rate was **49.2%**. In the multivariate analysis, elevated lactate dehydrogenase (LDH) (*p* =.004), ECOG performance status (PS) 2-4 (*p* =.006), and Ann Arbor stage III-IV (*p* =.008) affected OS. Stratification by elevated LDH, ECOG PS 2-4, and Ann Arbor stage III-IV allowed classification of patients into low risk, intermediate risk, and high risk groups. The same classification was applicable for HS and non-HS categories. In the rare neoplasms of histiocytic and dendritic cell sarcoma, ECOG PS, Ann Arbor stage, and LDH are important prognostic factors for predicting survival.

## INTRODUCTION

Histiocytic and dendritic cell neoplasms are extremely rare disorders, accounting for no more than 1% of malignant neoplasms occurring in the soft tissues and lymph nodes [1]. In the World Health Organization (WHO) 2008 classification, they are classified into histiocytic sarcoma (HS), Langerhans cell sarcoma (LCS), Langerhans cell histiocytosis (LCH), follicular dendritic cell sarcoma (FDCS), interdigitating cell sarcoma (IDCS), indeterminate dendritic cell sarcoma [1].

Various prognostic factors have been reported for hematological malignancies. The International Prognostic Index used for malignant lymphoma allows stratification of prognosis into 4 groups according to age, Eastern Cooperative Oncology Group (ECOG) Performance Status (PS), lactate dehydrogenase level (LDH), and Ann Arbor stage [2]. Similarly, the Revised International Prognostic Scoring System allows prognostic stratification of myelodysplastic syndrome according to risk factors, while the treatment strategy also differs depending on the number of risk factors [3].

To date, prognostic factors have been reported separately for different histiocytic and dendritic cell neoplasms. Specifically, in the case of HS, advanced stage, high MIB-1 (≥ 20%), and lesions greater than 3.5 cm are considered to indicate poor prognosis [4–6]. In LCH, multisystem disease indicates a poor prognosis, particularly when the lung, liver, spleen, or bone marrow is involved [7, 8]. For IDCS, advanced stage, younger age (≤ 40 years) and intra-abdominal invasion are considered to indicate poor prognosis [9].

BRAF is a phosphorylated protein associated with cellular transmission and cell growth via the RAS-ERK pathway. The BRAF*^V600E^* mutation is present in many malignant tumors, e.g., malignant melanoma [10]. This mutation leads to persistent activation of BRAF, and is affected by increasing tumor progression and changing tumor microenvironment [11]. The frequency of the BRAF*^V600E^* mutation was reported to be 35-57% in LCH [12–16] and 54% in Erdheim-Chester disease (ECD) [17].

The clinical characteristics, prognostic factors, and frequency of the BRAF*^V600E^* mutation in histiocytic and dendritic cell neoplasms have not been reported in a large number of cases. Here, we report a study of histiocytic and dendritic cell neoplasms in adults aged 18 years and over in Japan.

## RESULTS

### Clinical features

Table 1 summarizes the clinical data of the 87 cases.

**Table 1:** Clinical features of patients with histiocytic and dendritic cell neoplasms

	HS	LCH	FDCS	LCS	IDCS
	N=50	N=12	N=11	N=8	N=6
Age; median [range]	68 [21–88]	32.5 [18–71]	68.5 [31–71]	63 [45–74]	52.5 [33–78]
Sex: Male/Female	25/25	8/4	7/4	7/1	6/0
Clinical presentation					
Nodal lesion only	31.8% (14/44)	18.2% (2/11)	50.0% (5/10)	14.3% (1/7)	40.0% (2/5)
Extranodal lesion only	27.3% (12/44)	63.6% (7/11)	20.0% (2/10)	28.8% (2/7)	40.0% (2/5)
Nodal and extranodal lesion	40.9% 818/44)	18.2% (2/11)	30.0% (3/10)	57.1% (4/7)	20.0% (1/5)
Laboratory data					
Albumin≦3.5 g/dl	52.5% (21/40)	22.2% (2/9)	60.0% (6/10)	20.0% (1/5)	60.0% (3/5)
Elevated LDH level	54.5% (24/44)	18.2% (2/11)	20.0% (2/10)	28.6% (2/7)	40.0% (2/5)
Elevated CRP level	55.3% (21/38)	18.2% (2/11)	60.0% (6/10)	66.7% (4/6)	80.0% (4/5)
Clinical status					
ECOG PS2-4	42.2% (19/45)	10.0% (1/10)	20.0% (2/10)	42.9% (3/7)	0% (0/5)
B symptoms	31.1% (14/45)	27.3% (3/11)	10.0% (1/10)	28.6% (2/7)	40.0% (2/5)
Ann Arbor stage III-IV	68.9% (31/45)	36.4% (4/11)	60.0% (6/10)	71.4% (5/7)	60.0% (3/5)
Genetic alteration					
BRAF*^V600E^* mutation	6.1% (2/33)	0% (0/7)	0% (0/8)	0% (0/3)	20.0% (1/5)
Therapy					
Chemotherpay	18	4	4	3	1
Radiation therapy	4	0	1	0	1
Curative operation	8	2	3	0	1
Chemotherapy+ Radiation therapy	4	0	1	1	0
Chemotherapy+ Operation	1	0	0	0	0
No therapy	11	4	1	3	2
					
Follow up periods (months)	7 (0.1-119)	35 (1-80)	20 (1-88)	7.4 (0.5-30)	26 (1-52)

HS, Histiocytic sarcoma: LCH, Langerhans cell histiocytosis; FDCS, Follicular dendritic cell sarcoma: LCS, Langerhans cell sarcoma: IDCS, Interdigitating cell sarcoma: LDH, Lactate dehydrogenase: CRP, C-reactive protein: PS, Performance status.

**Figure 1:**
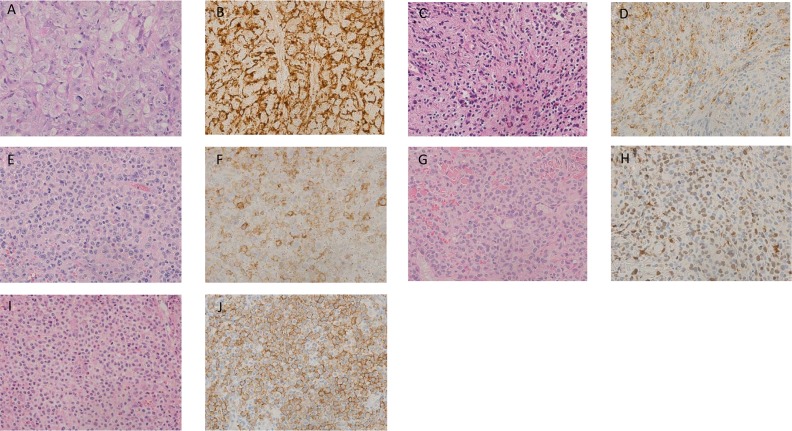
Histological findings of histiocytic and dendritic cell neoplasms **(A)** Histiocytic sarcoma: (hematoxylin and eosin [HE] staining, ×400). Large pleomorphic tumor cells show diffuse proliferation. Tumor cells have moderate to abundant eosinophilic cytoplasm. **(B)** Histiocytic sarcoma: Tumor cells positive for CD68 (KP-1) in the cytoplasm (×400). **(C)** Langerhans cell histiocytosis: (HE staining, ×400). Cytoplasm is clear to mildly eosinophilic. Nucleus has coffee bean-like nuclei and a nuclear ditch. The background shows infiltration of small lymphocytes and eosinophils. **(D)** Langerhans cell histiocytosis: Tumor cells positive for Langerin (CD207) in the membrane (×400). **(E)** Langerhans cell sarcoma: (HE staining, ×400). Polymorphic tumor cells with marked atypia and pleomorphic nuclei are observed. High mitotic activity are observed. **(F)** Langerhans cell sarcoma: Neoplastic cells positive for CD1a in the membrane (×400). **(G)** Interdigitating cell sarcoma: (HE staining, ×400). Proliferation of pleomorphic atypical cells is observed. Tumor cells show moderately to abundantly eosinophilic and clear cytoplasm. **(H)** Interdigitating cell sarcoma: Tumor cells positive for S-100 in the nuclei (×400). **(I)** Follicular dendritic cell sarcoma: (HE staining, ×400). Proliferation of atypical cells with admixed spindle- to ovoid-shaped nuclei is observed. Indistinct cytoplasmic borders and eosinophilic cytoplasm are observed. **(J)** Follicular dendritic cell sarcoma: Neoplastic cells positive for CAN.42 in the cytoplasm (×400).

Figure 1 shows pathological features of histiocytic and dendritic cell sarcoma.

#### Histiocytic sarcoma

Histiocytic sarcoma was diagnosed in 50 patients. The median age of these patients was 68 years (range: 21-88 years), and the male-to-female ratio was 1:1. Nodal involvement was noted in 31.8% (14/44), extranodal involvement in 27.3% (12/44), and both nodal and extranodal involvement in 40.9% (18/44) of these patients. Elevated LDH was noted in 54.5% (24/44 cases). In total, 42.2% of patients (19/45) had ECOG PS 2-4, and 68.9% (31/45) of patients were at an Ann Arbor stage III-IV. The frequency of *IGH-BCL2* rearrangement was 0% (0/10). The median follow-up period was 7 months (range: 0.1-119 months). Chemotherapy was administered to 50.0% (23/46) of patients. The CHOP regimen, composed of doxorubicin, cyclophosphamide, vincristine, and prednisone was the most common (47.8%; 11/23 cases) regimen used. The other patients received radiation therapy (8 cases), curative operation (8 cases), or no therapy (11 cases). In total, 58.7% (27/46) of the patients died of the original disease.

#### Langerhans cell histiocytosis

LCH was noted in 12 patients. Their median age was 32.5 years (range: 18-71 years), and the male-to-female ratio was 2:1. Seven patients had single-system disease, with a single site involved in 5 cases and multiple sites in 2 cases. The organ involved was the skin in 2 cases, bone in 2 cases, lymph node in 2 cases, and the pleura in 1 case. Two patients had multisystem disease. The organs involved were the pituitary gland, skin, bone, lymph nodes, and/or spleen. Elevated LDH was present in 18.2% (2/11) of the patients. ECOG PS, 2-4 was observed in 10.0% (1/10) of the patients, and 36.4% (4/11) of the patients were at an Ann Arbor stage III-IV. The frequency of positivity for CD56 was 9.1% (1/11).

The median follow-up period was 35 months (range: 1-80 months). Chemotherapy was administered to 40.0% (4/10) of patients, including the JLSG02 [18] protocol (3 cases) and methotrexate (1 case). The other patients received curative operation (2 cases) or no therapy (4 cases). In total, 18.2% (2/11) of patients died due to a deterioration of the original disease.

#### Langerhans cell sarcoma

Langerhans cell sarcoma was diagnosed in 8 patients. Their median age was 68.5 years (range: 31-87 years); the male-to-female ratio was 7:1. Three patients had a single-system disease, with a single site in 2 cases and multiple sites in 1 case. The organs involved were the skin (2 cases) and lymph nodes (1 case). Four patients had multisystem disease. The organs involved were lymph nodes, skin, bone, spleen, and bone marrow. Elevated LDH was noted in 28.6% (2/7) of patients. ECOG PS 2-4 was observed in 42.9% of (3/7) patients, and 71.4% (5/7) of patients were at an Ann Arbor stage III-IV. The frequency of *IGH-BCL2* rearrangement was 0% (0/3). The frequency of positivity of CD56 was 25.0% (1/4).

The median follow-up period was 7.4 months (range: 0.5-30 months). Chemotherapy was administered to 57.1% (4/7) of the patients. The JLSG02 protocol (1 case) and CHOP regimen (3 cases) were primarily used. The other patients received no therapy (3 cases). A total of 57.1% (4/7) of patients died due to deterioration of the original disease.

#### Follicular dendritic cell sarcoma

Follicular dendritic cell sarcoma was noted in 11 patients. Their median age was 63 years (range: 45-74 years); the male-to-female ratio was 1.75:1. Fifty percent (5/10) of patient demonstrated only nodal involvement, 20% (2/10) showed extranodal involvement only, and 30% (3/10) showed both nodal and extranodal involvement. Elevated LDH was noted in 20.0% (2/10), ECOG PS 2-4 in 20.0% (2/10), and Ann Arbor stage III-IV in 60.0 % (6/10) of patients.**The frequency of EBER-positive cases was 36.4% (4/11)**.

The median follow-up period was 20 months (range: 1-88 months). Chemotherapy was administered to 50.0% (5/10) of patients. The CHOP regime (3 cases), prednisone (1 case), and gemcitabine plus dexamethasone (1 case) were used. The other 5 patients received curative operation (3 cases), radiation therapy (1 case), or no therapy (1 case). Fifty percent (5/10) of patients died of the original disease.

#### Interdigitating cell sarcoma

Interdigitating cell sarcoma was diagnosed in 6 patients. The median age was 52.5 years (range: 33-78 years); the male-to-female ratio was 6:0. Of these, 40.0% (2/5) showed only nodal involvement and 40.0% (2/5) showed only extranodal involvement. Forty percent had elevated LDH (2/5 cases). ECOG PS 2-4 was noted in 0% (0/5) of cases, and 60.0% (3/5) were at an Ann Arbor stage III-IV. The median follow-up period was 26 months (range: 1-52 months). Chemotherapy was administered to 20.0% (1/5) of patients (details unknown). The others received radiation therapy (1 case), curative operation (1 case), or no therapy (2 cases). Twenty percent (1/5) of patients died from the original disease.

### Analysis of BRAF^V600E^ mutation

Data for 57 patients (65.5%, 57/87) were analyzed for the BRAF*^V600E^* mutation; these included 33 HS cases, 7 LCH cases, 8 FDCS cases, 3 LCS cases, 5 IDCS cases, and 1 indeterminate dendritic cell sarcoma case. The mutation was present in 3 of 57 cases (5.3%), including 2 of 33 HS cases (6.1%) and 1 of 5 IDCS cases (20.0%).

### Survival analysis

**Figure 2:**
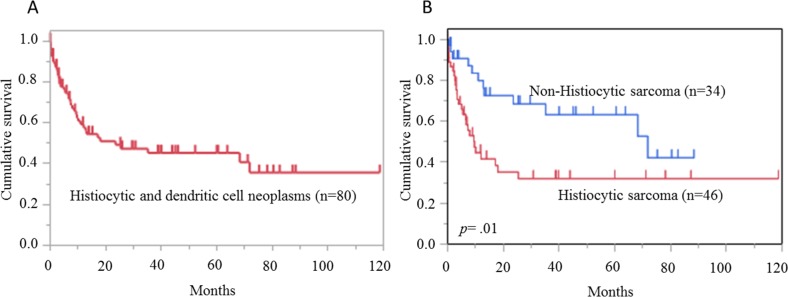
Overall survival in histiocytic and dendritic cell neoplasms **(A)** Overall survival of 80 cases of histiocytic and dendritic cell neoplasms. Median follow-up period and median survival time were 18.0 months and 23.5 months, respectively. **(B)** The comparison of overall survival curves between histiocytic sarcoma (HS) and non-HS. HS shows significantly worse prognosis (*p* =.01).

Clinical data were obtained for 80 patients (92.0 %) with histiocytic and dendritic cell neoplasm; 39 patients (48.8 %) died of the original disease (Figure 2A). The median follow-up period was 18.0 months (range: 9.6-71.8 months), and the median OS was 23.5 months. The OS rate was 49.2% at 2 years. There was a statistically significant difference between the OS curves of the HS and non-HS groups (*p* =.01) (Figure 2B). The non-HS group included 11 patients with LCH, 7 of LCS, 10 of FDCS, and 5 of IDCS. The OS for the various subtypes is shown in Supplementary Figure 1.

### Univariate analysis and multivariate analysis

Univariate and multivariate analysis results of OS for histiocytic and dendritic cell neoplasms are shown in Table 2. In univariate analysis, albumin ≤ 3.5 g/dl (hazard ratio [HR] 2.33; 95% confidence interval [CI] 1.13-4.79; *p* =.02), elevated CRP level (HR 2.95; 95% CI 1.39-6.27; *p* =.005), elevated LDH level (HR 4.46; 95% CI 2.20-9.03; *p* <.0001), B symptoms (HR 2.06; 95% CI 1.04-4.06; *p* =.04), ECOG PS, 2-4 (HR 4.64; 95% CI 2.36-9.13; *p* <.0001), extranodal lesions (HR 2.27; 95% CI 1.06-4.83; *p* =.03), and Ann Arbor stage III-IV (HR 7.59; 95% CI 2.94-19.61; *p* <.0001) were significant factors in OS.

**Table 2:** Univariate and multivariate analysis for overall survival of patients with histiocytic and dendritic cell neoplasms

	Parameters	Hazard ratio [95% confidence interval]	*p*-value
**Univariate analysis**			
	Albumin ≤3.5 g/dl	2.33 [1.13-4.79]	.02
	Elevated LDH level	4.46 [2.20-9.03]	<.0001
	Elevated CRP level	2.95 [1.39-6.27]	.005
	Age >40 years	3.43 [0.82-14.28]	.09
	B symptoms	2.06 [1.04-4.06]	.04
	ECOG PS 2-4	4.64 [2.36-9.13]	<.0001
	Lymph node invasion	1.37 [0.69-2.75]	.37
	Extranodal invasion	2.27 [1.06–4.83]	.03
	Intra-abdominal lesions	1.57 [0.83–2.97]	.17
	≥3.5 cm lesions	1.18 [0.61–2.26]	.62
	Ann Arbor stage III-IV	7.59 [2.94–19.61]	<.0001
	Received therapy	0.85 [0.43–1.68]	.65
**Multivariate analysis**			
	Albumin ≤3.5 g/dl	2.70 [1.09–6.72]	.003
	Elevated LDH level	3.27 [1.47–7.26]	.004
	ECOG PS 2-4	3.39 [1.43–8.07]	.006
	Ann Arbor stage III-IV	5.27 [1.56–17.82]	.008

LDH, Lactate dehydrogenase: CRP, C-reactive protein: PS, Performance status

In multivariate analysis, albumin ≤ 3.5 g/dl (HR 2.70; 95% CI 1.09-6.72; *p* =.003), elevated LDH level (HR 3.27; 95% CI 1.47-7.26; *p* =.004), ECOG PS 2-4 (HR 3.39; 95% CI 1.43-8.07; *p* =.006), and Ann Arbor stage III-IV (HR 5.27; 95% CI 1.56-17.82; *p* =.008) were significant factors in OS.

### Stratification of OS by prognostic indexes

**Figure 3:**
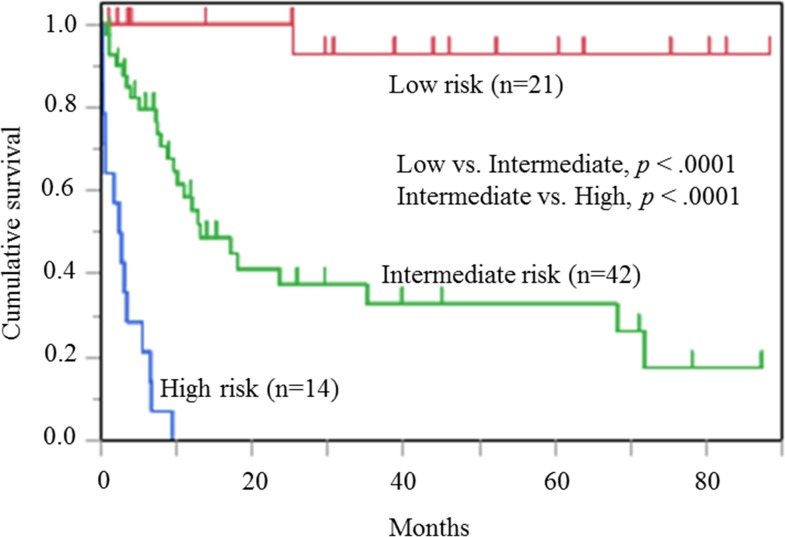
Risk classification of patients with histiocytic and dendritic cell neoplasms Classification of 77 cases with histiocytic and dendritic cell neoplasms into 3 risk groups (Low risk, Intermediate risk, and High risk) according to elevated LDH, ECOG PS, 2-4, and Ann Arbor stage III-IV. There are significant differences between groups next to each other: Low risk group vs. Intermediate risk group, *p* <.0001; Intermediate risk group vs. High risk group: *p* <.0001.

Stratification was performed using the 3 factors that showed a significant difference in multivariate analysis: ECOG PS 2-4, Ann Arbor stage III-IV, and elevated LDH. The low risk group had none of the 3 factors, the intermediate risk group had any 1 or 2 of these factors, and the high risk group had all 3 factors. The stratification results are shown in Figure 3.

Twenty-one cases were in the low risk group (27.3%; median survival: NA months); 42 cases were in the intermediate risk group (54.5%; median survival, 13.1 months; 95% CI 9.60-35.17 months); and 14 cases were in the high risk group (18.1 %; median survival, 2.52 months; 95% CI 0.27-5.43 months). The score allowed stratification of patient prognoses (low risk group vs. intermediate risk group, *p* <.0001; intermediate risk group vs. high risk group, *p* <.0001).

Hazard ratio (HR) for overall survival of patients with histiocytic and dendritic cell neoplasms in 3 risk groups is shown in Table 3. The intermediate risk group and high risk group showed significantly worse OS than the low risk group (HR 12.0; 95% CI 1.741-82.722, and HR 21.0; 95% CI 3.101-142.202, respectively).

**Table 3:** Hazard ratio for overall survival of patients with histiocytic and dendritic cell neoplasms in 3 risk groups

Risk group	Hazard ratio	[95% confidence interval]	*p*-value
**Low risk group**	1		
**Intermediate risk group**	12.0	1.741-82.722	<.0001
**High risk group**	21.0	3.101-142.202	<.0001

### Stratification of OS by prognostic indexes for HS and non-HS

**Figure 4:**
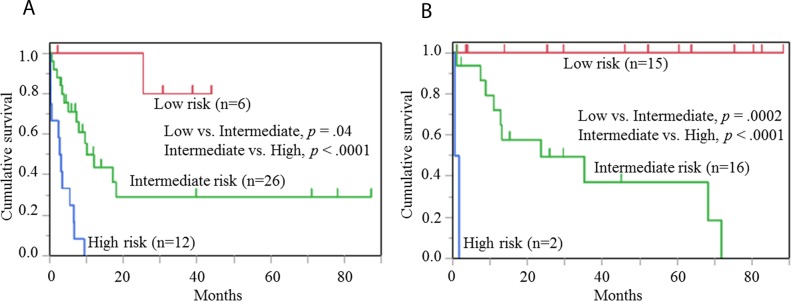
Risk classification of cases with histiocytic and non-histiocytic sarcomas **(A)** Classification of 44 cases with histiocytic sarcoma into 3 risk groups. There are significant differences between groups next to each other: Low risk group vs. Intermediate risk group, *p* =.04; Intermediate risk group vs. High risk group, *p* <.0001. **(B)** Classification of 36 cases with non-histiocytic sarcoma into 3 risk groups. There are significant differences between groups next to each other: Low risk group vs. Intermediate risk group, *p* =.0002; Intermediate risk group vs. High risk group, *p* <.0001.

Figure 4A and 4B show the results of analysis of the 3 risk groups, separately, for HS and non-HS. Similar stratification was possible for the HS group (low risk group vs. intermediate risk group, *p* =.04; intermediate risk group vs. high risk group, *p* <.0001) and the non-HS group (low risk group vs. intermediate risk group, *p* =.0002; intermediate risk group vs. high risk group, *p* <.0001).

## DISCUSSION

We analyzed histiocytic and dendritic cell neoplasm patients to determine the clinical characteristics, prognostic factors, and frequency of the BRAF*^V600E^* mutation in a large number of cases. Our multivariate analysis revealed 3 factors, ECOG PS 2-4, Ann Arbor stage III-IV, and elevated LDH as factors involved in poor prognosis, which allowed patients to be classified into 3 risk groups.

The clinical data from previous reports containing at least 5 cases of HS, a total of 58 cases [4,6,19–21] are shown in Supplementary Table 1. The clinical characteristics of the current and previous patients did not differ markedly, and indicate that HS is a neoplasm with a poor prognosis.

The prognosis for adult LCH is reportedly better than that in infants [22, 23]. In a study of 274 cases of adult LCH, the 5-year OS was 92.3%, and mortality rate was 6.4% (15/236 cases) [24]. In our study, 1 of 9 LCH cases died due to the original disease in 71.8 months.

In a previous review of 66 LCS cases, the median OS was 27.2 months [25], whereas the median OS for LCS patients in our study was 7.4 months. The reasons for this discrepancy was unclear, but may be related to difference in the proportion of cases with multisystem disease (71.4% [5/7 cases] in the present study vs. 40.9% [27/66 cases] in the previous study).

CD56 has been reported as a poor prognostic marker in LCH and LCS, [26, 27]. In our study, positivity for CD56 in LCH and LCS were 9.1% (1/11) and 25.0% (1/4), respectively. There was no clinicopathological difference between CD56-positivity and -negativity, and it was not associated with a poor prognosis, although it was difficult to examine the relationship with prognosis, since there were only 4 cases with LCS.

In this study, 36.4% (4/11 cases) of FDCS patients were diagnosed with Inflammatory pseudo tumor (IPT)-like FDC sarcoma. IPT-like FDC sarcoma often tests positive for EBER-ISH [28]. In our EBER-ISH analysis in FDCS, all cases that were EBER-ISH-positive (36.1%:4/11) were IPT-like FDCS.

About 27% cases of IPT-like FDC sarcoma in previous reports had splenic lesions [28]. All cases in this study developed splenic lesions. In all cases, splenectomy was performed and survived without treatment during the follow up period (median 82.6 months [25.2-88.4 months]). This seems to be consistent with a report that disease-free survival in cases with IPT-like FDC sarcoma is better than in cases with typical FDC sarcoma [28].

In a previous report of 66 cases of FDCS [29], extra-nodal disease and bulky or intra-abdominal disease at presentation were associated with a poor prognosis. However, advanced stage was not a prognostic factor. In contrast, Gounder et al. have reported that advanced stage was a poor prognostic factor in 31 cases of FDCS [21]. In our study, advanced stage was one of the poor prognostic factors. However, in our study, only 11 cases of FDCS were included; it is therefore necessary to investigate many more cases of FDCS in future.

We were able to classify cases of histiocytic and dendritic cell neoplasms into 3 risk groups by scoring the Ann Arbor stage III-IV, elevated LDH, and ECOG PS 2-4. In previous reports of HS [4–6], MIB-1 ≥ 20%, a lesion of greater than 3.5 cm, and advanced stage have been reported to indicate a poor prognosis. Multisystem disease implied a poor prognosis in LCH; invasion of the lung, liver, spleen, and bone marrow were considered to indicate a particularly poor prognosis [7, 8]. For IDCS, advanced stage, age ≤ 40 years, and intra-abdominal invasion are said to indicate poor prognosis [9]. To date, in many previous reports [4, 5, 7, 8] that have studied prognostic factors of different disorders among the histiocytic and dendritic cell neoplasms, advanced stage was a common poor prognostic factor, as also found in this study (*p* <.0001). However, elevated LDH and ECOG PS 2-4 cited here as prognostic factors have rarely been included in analysis in the many previous reports [4, 5, 7, 8]. Therefore, stratification based on the risk factors of Ann Arbor stage III-IV, elevated LDH, and ECOG PS 2-4 could constitute a new prognostic index for histiocytic and dendritic cell neoplasms.

Our study had some limitations. The prognostic factors were not examined in a population receiving uniform treatment, and thus, it was not possible to study therapeutic effects. Moreover, as histiocytic and dendritic cell neoplasm is a heterogenous disease, it is unclear whether this model applies to all subtypes. In particular, there were few cases of LCS and IDCS among other subtypes, in our study, and additional studies including many cases of the various subtypes are required.

In this study, 6.1% (2/33) of patients with HS carried the BRAF*^V600E^* mutation. While, in a study from Korea, 62.5% (5/8) of HS patients carried the BRAF*^V600E^* mutation [30]. It has been reported that age may greatly influence the frequency of the BRAF*^V600E^* mutation in LCH, as it was more frequently found in children than in adults [31, 32]. Our study examined HS only in adult cases, but in the study from Korea [30], pediatric cases were included among the BRAF*^V600E^* mutation cases. Even in HS, it is possibly that age may be involved in the prevalence of the BRAF*^V600E^* mutation. On the other hand, in studies reporting 3 cases [17] and 5 cases [33] of HS from western countries, the BRAF*^V600E^* mutation was absent. Mutations other than the BRAF*^V600E^* were also found in BRAF in 60% (3/5) of cases in one study [33]. In our study, we have not searched for mutations other than the BRAF*^V600E^*, and it is possible that other mutations, such as the BRAF*^G464V^* and BRAF*^G466R^* in exon 11 could be involved.

In conclusion, we showed that using the 3 factors of ECOG PS 2-4, Ann Arbor stage III-IV, and elevated LDH allowed classification of patients with histiocytic and dendritic cell neoplasm into 3 risk groups.

## MATERIALS AND METHODS

### Patient characteristics

**Eighty-seven cases** of histiocytic and dendritic cell neoplasms in subjects aged ≥ 18 years, diagnosed by the Pathology Department of Kurume University, Japan, from 2005 to 2016, were studied. All cases were reviewed by hematopathologists (KO and HM) and diagnosed according to the WHO classification [1]. Clinical and survival data were obtained for 80 cases. Information regarding age, sex, B symptoms, performance status, serum albumin, serum LDH, serum C-reactive protein (CRP) level, Ann Arbor stage, and the presence of nodal and extranodal lesions was obtained by reviewing the patients’ medical charts. The Ann Arbor classification was used for staging in a previous report [19]. For LCH/LCS, classification into single-system disease or multiple system disease is commonly used [34]. Single-system disease is defined as an Ann Arbor stage I-II, and multiple system disease as an Ann Arbor stage III-IV. The use of materials and clinical information was approved by the Research Ethics Committee of Kurume University and was in accordance with the Declaration of Helsinki.

### DNA extraction, polymerase chain reaction (PCR) analysis, and Sanger sequencing of BRAF

Genomic DNA was extracted using a QIAamp DNA FFPE Tissue Kit (Qiagen, Hilden, Germany). Primer sets used for PCR and sequencing analyses are summarized in Supplementary Table 2. Mutation analyses were performed as previously described [35].

#### Fluorescence in situ hybridization (FISH) analysis

FISH was performed for *IGH-BCL2* translocation (KBI-10606; Leica Microsystems) as previously described [36].

### Statistical analysis

Survival curves of overall survival (OS) were calculated by the Kaplan–Meier method. The end-points of OS were defined as the time of death due to histiocytic and dendritic neoplasms. A log-rank test was used to compare survival curves. Univariate and multivariate analyses were performed to evaluate the influence of prognostic factors on the OS by the Cox proportional hazards models. The statistical analyses were carried out in JMP, version 10 (SAS institute, Tokyo, Japan). *P*-values < 0.05 indicated statistical significance.

## SUPPLEMENTARY MATERIALS FIGURES AND TABLES



## Abbreviations

HS: Histiocytic sarcoma
LCS: Langerhans cell sarcoma
LCH: Langerhans cell histiocytosis
FDCS: follicular dendritic cell sarcoma
IDCS: Interdigitating cell sarcoma
JXG: juvenile xanthogranuloma
ECOG PS: Eastern Cooperative Oncology Group performance status
LDH: Lactate dehydrogenase
ECD: Erdheim-Chester disease
CRP: C-reactive protein
OS: Overall survival
HR: Hazard ratio
CI: Confidence interval
