# Supplementary material for: Prognostic factors for histiocytic and dendritic cell neoplasms
Source: Oncotarget. 2017 Oct 19;8(58):98723–32. doi: 10.18632/oncotarget.21920 (PMC5716762; doi:10.18632/oncotarget.21920)
Supplement: Supplementary file 1 [file oncotarget-08-98723-s001.pdf]

Prognostic factors for histiocytic and dendritic cell neoplasms

SUPPLEMENTARY MATERIALS

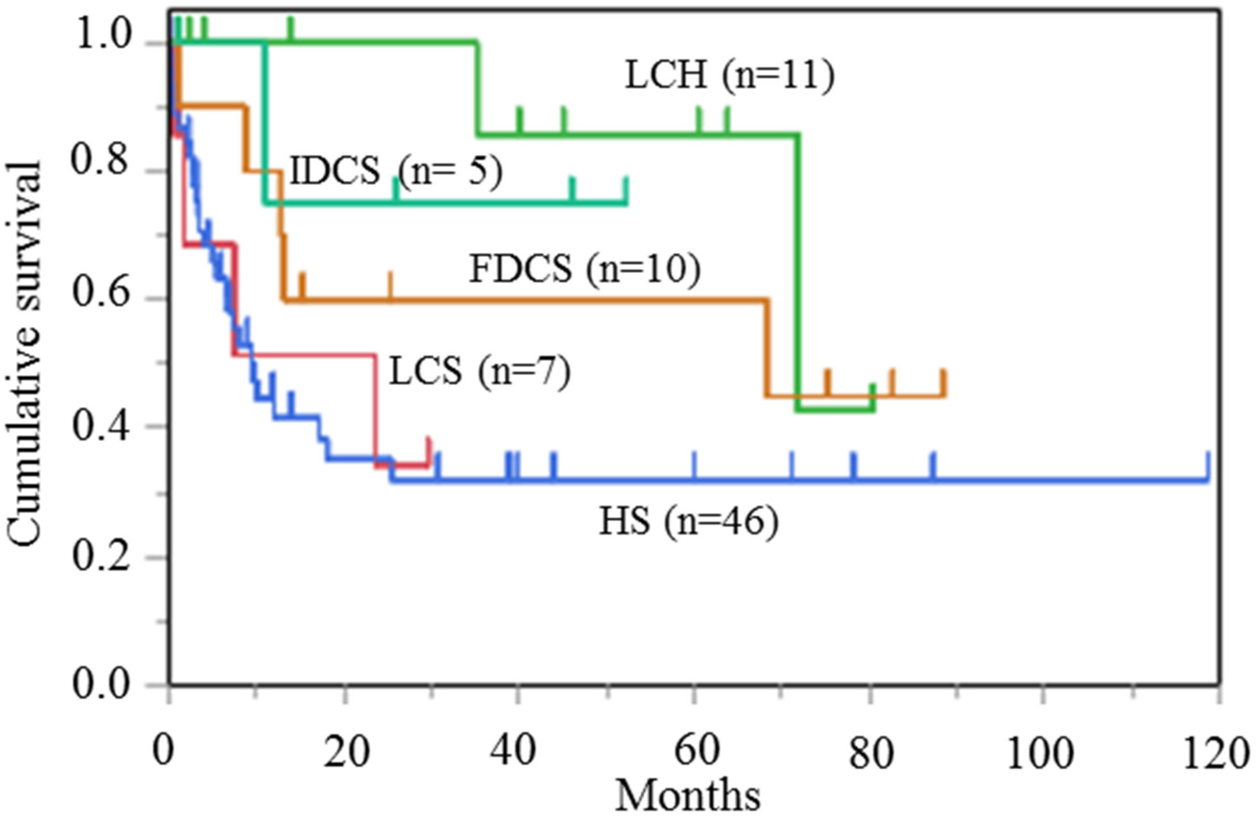

HS	46	11	6	5	3	2
LCS	7	3	0	0	0	0
LCH	11	7	5	3	0	0
FDCS	10	5	4	4	2	0
IDCS	5	3	2	0	0	0

Supplementary Figure 1: Overall survival for each subtype: histiocytic sarcoma (HS), Langerhans cell histiocytosis (LCH), Langerhans cell sarcoma (LCS), follicular dendritic cell sarcoma (FDCS), and interdigitating cell sarcoma (IDCS). The 2-year OS rates were 34.3% for HS, 100% for LCH, 35.2% for LCS, 60.0% for FDCS, and 75.0% for IDCS.

Supplementary Table 1: Comparison between our study and previous reports of Histiocytic sarcoma

	Our study N = 50	Previous reports* N = 58
**Age: median [range]**	68 [21-88]	56 [0.5-89]
**Sex: Male/Female**	1/1	1.4/1
**Clinical presentation**		
**Nodal lesion only**	31.8% (14/44)	11.8% (4/34)
**Extranodal lesion only**	27.3% (12/44)	44.1% (15/34)
**Nodal and extranodal lesion**	40.9% (18/44)	44.1% (15/34)
**Laboratory data**		
**Albumin ≤ 3.5 g/dl**	52.5% (21/40)	NA
**Elevated LDH level**	54.5% (24/44)	NA
**Elevated CRP level**	55.3% (21/38)	NA
**Clinical status**		
**ECOG performance status 2-4**	42.2% (19/45)	NA
**B symptoms**	31.1% (14/45)	50.0% (6/12)
**Ann Arbor Stage III-IV**	68.9% (31/45)	47.8% (22/46)
**Therapy**		
**Chemotherapy**	18	23
**Radiation therapy**	4	4
**Curative operation**	8	13
**Chemotherapy+ radiation therapy**	4	3
**Chemotherapy+ operation**	1	2
**Radiation therapy+ operation**	0	0
**No therapy**	11	4
**Follow up periods (months)**	7 (0.1-119)	26 (0.1-221)
**Dead due to disease**	58.7% (27/46)	45.2% (19/42)

*Previous reports: *Cancers (Basel)* 2014; 6: 2275-95 (Ref. 4); *Mod Pathol* 2005; 18: 693-704 (Ref 6); *Histopathology* 2002; 41: 1-29 (Ref 19); *Am J Surg Pathol* 2004; 28: 1133-44 (Ref 20), and *Eur J Cancer* 2015; 51: 2413-22 (Ref 21).
LDH, Lactate dehydrogenase: CRP, C-reactive protein: NA, Not available

Supplementary Table 2: Primer sets used for sequencing analyses for BRAF mutation in our study

Primer	Sequence (5'-3')
**BRAF-Forward- first step PCR**	CATAATGCTTGCTCTGATAGGAAAATGAG
**BRAF Reverse- first step PCR**	AACTCAGCCAGCATCTCAGGGCCAA
**BRAF-Forward - second step PCR**	CCTTTACTTACTACACCTCAGATATATTC
**BRAF Reverse- second step PCR**	GGAAAAATAGCCTCAATTCTTACCATCCAC
